# Editorial: Digital health technologies for shared decision making

**DOI:** 10.3389/fdgth.2025.1574102

**Published:** 2025-05-07

**Authors:** Marcus Cheetham, Helena Canhão, Anna Lisa Martin-Niedecken, Nikola Biller-Andorno, Christoph A. Meier

**Affiliations:** ^1^ Department of Internal Medicine, University Hospital Zurich, Zurich, Switzerland; ^2^ Comprehensive Health Research Center, New University of Lisbon, Lisbon, Portugal; ^3^ Institute for Design Research, Department of Design, Zurich University of the Arts, Zurich, Switzerland; ^4^ Institute of Biomedical Ethics and History of Medicine, University Zurich, Zurich, Switzerland

**Keywords:** patient decision aids (PtDAs), shared decision making (SDM), digital health (DH), artificial intelligence (AI), human factors, values, preferences, design

**Editorial on the Research Topic**
Digital health technologies for shared decision making

Current and emerging digital health technologies (DTHs) present a broad spectrum of opportunities to support and advance traditional approaches to shared decision making (SDM). In this evolving field, a key challenge lies in discerning where, when and how to best utilize DTHs to enhance SDM-related processes and outcomes. The articles in this Research Topic *“Digital Health Technologies for Shared Decision Making”* present a snapshot of current developments and viewpoints on this challenge.

DHTs can help to understand, reach and support patients across their healthcare journey (1–3). Höppchen et al. consider how suitably designed DHTs can target and leverage human factors to improve patient engagement. Based on the use case of cardiac rehabilitation, the authors examine barriers and facilitators to patient engagement across the stages of healthcare from awareness to SDM. They present the implications of their findings for the design and implementation of DTHs.

Within the healthcare journey, complex treatment pathways can present various key moments for SDM at which patient preferences, values and experiences can significantly influence the course of treatment (4). With a focus on intensive care, Göcking et al. apply patient journey mapping to identify and generate a structured overview of preference-sensitive moments during treatment at which timely engagement of patients can aid preparation, facilitation and reflection about shared decisions. The authors consider the strategic implementation of DTHs at these moments to align patient care with patient needs, values and preferences.

One strategy for facilitating alignment between patient care and their needs, values and preferences is the use of Patient Decision Aids (PtDAs). These aids aim to help patients better prepare for and participate in the SDM consultation (5). While digital capabilities can facilitate this aim, they also present challenges in PtDA design and use (6). With a focus on PtDAs for treatment selection in depression, Sedlokova et al. identify, evaluate and compare the strengths and weaknesses (in terms of e.g., accessibility, information design, personalization, adaptability) of analogue and digital PtDAs in relation to their effectiveness in promoting patient engagement in SDM.

Human factors, like depression and anxiety, can influence how patients process information and decisional situations, impacting the design considerations for digital health tools (7). Depression and anxiety are associated with altered patterns of risk perception, involvement in decision making and experience of decisional conflict in SDM (8, 9). Fanio et al. report on the unique challenges of designing a PtDA for anxious patients. With a focus on atrial fibrillation, the authors consider the incorporation of specific design features to facilitate a supportive digital environment with which to mitigate effects of anxiety on information and decision making.

A key strategy for effective SDM is to support collaboration between patients and healthcare professionals (10). Wurhofer et al. examine the practical application of a digital tool for collaborative planning in cardiac rehabilitation and its impact on SDM. Based on their findings, the authors identify opportunities for supporting collaboration before, during and after SDM and consider the digital implementation of corresponding design features to facilitate SDM (i.e., *SDM-supportive design*).

*Artificial intelligence* (*AI*) and DHT have the potential to enhance SDM in different ways. Early studies can provide important insight to shape further development and refinement of AI in SDM. Singh et al. focus on orthopaedic practice in an early phase translational design, feasibility and usability study. They develop and evaluate an interactive approach for integrating knowledge of patient preferences and priorities into the SDM consultation. The authors consider this approach in the context of informing the development of an AI-based personalized Health Recommender System for SDM.

Eiskjaer et al. consider a different AI-based approach to generating personalized patient support in SDM. Based on spinal disorders, the authors present a tool that applies predictive analytics to generate evidence-based insights into a patient's treatment options and the likely outcomes of these. These insights are used in SDM to personalize and encourage collaborative dialogue about these options. The authors consider factors that can drive or hinder the use of this tool for SDM.

To facilitate collaborative dialogue in SDM, Lin et al. evaluate an opponent model-based approach to SDM. This model simulates the interactive process in which a patient's initially vague preferences are distilled into more actionable insights as a patient engages in collaborative dialogue with their physician, gains clarity about their preferences, and reaches more informed and confident decisions. The authors examine this model in the context of developing treatment plans that fit individual preferences and consider its relevance for future application in SDM.

The integration of AI-enhanced DHTs in SDM raises sensitive and ethically challenging issues. Based on assisted suicide, Spitale et al. create AI models to extract and classify patient case reports from real-world data. The authors take these reports as a basis for examining the potential feasibility, challenges and dilemmas of using AI to help physicians navigate complex ethical issues about patient care, confidentiality and professional responsibility.

In summary, the preceding contributions seek to develop an evidence-led understanding of when, where and how DHTs can facilitate effective support for SDM. The diverse foci of these contributions hint at a broad range of potentially unmet needs and insufficiencies in SDM across multiple areas of healthcare and within the patient healthcare journey that suitably designed and implemented DHTs might help to address. Together, these contributions are also illustrative of the complexities of tailoring DHTs to diverse human factors relevant for effective engagement in SDM while integrating DHTs in the broader context of traditional SDM solutions and the healthcare practices, workflows and environments in which SDM is or could be situated. With these challenges in mind, research ranging from early conceptual thinking to mature technical developments and the evaluation of the effectiveness of existing and emerging DHTs for supporting SDM are needed (11). This research could lead to the formation of a body of practical design and implementation knowledge about ways in which DTHs can enhance SDM-related processes and outcomes.
